# The Role of Tissue Resident Memory CD4 T Cells in Herpes Simplex Viral and HIV Infection

**DOI:** 10.3390/v13030359

**Published:** 2021-02-25

**Authors:** Thomas R. O’Neil, Kevin Hu, Naomi R. Truong, Sana Arshad, Barbara L. Shacklett, Anthony L. Cunningham, Najla Nasr

**Affiliations:** 1 Centre for Virus Research, The Westmead Institute for Medical Research, Westmead, NSW 2145, Australia; thomas.oneil@sydney.edu.au (T.R.O.); kevin.hu@sydney.edu.au (K.H.); naomi.truong@sydney.edu.au (N.R.T.); sana.arshad@sydney.edu.au (S.A.); 2 Westmead Clinical School, Faculty of Medicine and Health, The University of Sydney, Westmead, NSW 2145, Australia; 3 Department of Medical Microbiology and Immunology, School of Medicine, University of California, Davis, CA 95616, USA; blshacklett@ucdavis.edu; 4 School of Medical Sciences, Faculty of Medicine and Health, The University of Sydney, Sydney, NSW 2000, Australia

**Keywords:** HIV-1, HSV-1/2, tissue resident CD4^+^, CD8^+^, vaccines, infection, immunity, keratitis

## Abstract

Tissue-resident memory T cells (TRM) were first described in 2009. While initially the major focus was on CD8^+^ TRM, there has recently been increased interest in defining the phenotype and the role of CD4^+^ TRM in diseases. Circulating CD4^+^ T cells seed CD4^+^ TRM, but there also appears to be an equilibrium between CD4^+^ TRM and blood CD4^+^ T cells. CD4^+^ TRM are more mobile than CD8^+^ TRM, usually localized deeper within the dermis/lamina propria and yet may exhibit synergy with CD8^+^ TRM in disease control. This has been demonstrated in herpes simplex infections in mice. In human recurrent herpes infections, both CD4^+^ and CD8^+^ TRM persisting between lesions may control asymptomatic shedding through interferon-gamma secretion, although this has been more clearly shown for CD8^+^ T cells. The exact role of the CD4^+^/CD8^+^ TRM axis in the trigeminal ganglia and/or cornea in controlling recurrent herpetic keratitis is unknown. In HIV, CD4^+^ TRM have now been shown to be a major target for productive and latent infection in the cervix. In HSV and HIV co-infections, CD4^+^ TRM persisting in the dermis support HIV replication. Further understanding of the role of CD4^+^ TRM and their induction by vaccines may help control sexual transmission by both viruses.

## 1. Introduction

The ability of our immune system to develop memory B and T cells is integral for long-term protection against invading pathogens and is the key rationale underpinning vaccinations. Originally T cells were thought to be primarily recirculating between blood and secondary lymphoid tissues. Recently, studies have shown that tissue compartments accumulate a large pool of resident CD4^+^ and CD8^+^ T cells that are transcriptionally, phenotypically, and functionally distinct from circulating T cells and within different tissues [1,2,3]. Tissue-resident memory (TRM) T cells are key targets in vaccination strategies as they mediate local immune responses [4] or directly interact with other immune cells [5]. The role of CD4^+^ helper T cells (Th) in response to pathogens is highlighted by their ability to aid and direct CD8^+^ T cells, B cells and innate immune cells. Additionally, CD4^+^ T cells have cytotoxic effects, particularly against HIV [6,7]. While memory T cells can recirculate between blood and tissues to deliver a quicker and more robust response upon re-exposure to the same pathogen, the TRM cells are poised to enhance this role as they are located at the entry portals for pathogens. However, their persistence in tissue after pathogen clearance, their mechanism of maintenance, and the properties that distinguish TRM in different tissues from circulating effector memory T cells (TEM) are still unclear.

Sexually transmitted diseases, such as human immunodeficiency virus (HIV) and herpes simplex virus (HSV), cross physical and immunological barriers to establish life-long, latent infections. There is an intimate interaction between HSV and HIV in this setting. Prior HSV2 infection enhances the risk of HIV acquisition at least three-fold and accounts for up to 50% of genital herpes in some regions [8]. Studies have pointed to mucosal CD4^+^ T cells as playing a role in controlling genital HSV infection and disease and are key candidate target cells for vaccine development. CD4^+^ T cells are also the primary target cells for HIV, and recently TRMs were shown to be more infected than their blood counterparts. Overall, both CD4^+^ and CD8^+^ TRMs are still largely understudied in the human genital mucosa. Therefore, we highlight knowledge gaps.

This review will focus on CD4 TRMs in view of the recent discoveries on their relative role in HSV and HIV infections and their under-investigation compared to murine CD8 and CD4 TRMs. In this context, we will summarise the ontogeny of CD4^+^ and CD8^+^ T cells, their identification, maintenance and their contributions to the local immune environment. We will also discuss their role in HSV and HIV infection, how HSV infection increases the risk of HIV acquisition, and how CD4^+^ TRMs in the genital tract could be used to enhance therapeutic strategies or contribute to vaccine development in the absence of an effective vaccine against HIV or HSV.

## 2. CD4^+^ T Cells in Blood

### 2.1. Development of T Cells

The common lymphoid precursors migrate from the bone marrow to the thymus to undergo the process of T cell maturation involving three steps of thymic selection [9,10,11]. Following the development of CD4^+^ CD8^+^ cells, T cell receptor (TCR) rearrangement occurs to select for functional, single-positive CD4^+^ or CD8^+^ TCRs. Cells displaying inadequate TCR and co-receptor signalling are removed via apoptosis as they do not bind to B cells and antigen-presenting cells (dendritic cells (DCs) and macrophages (Mfs)) with adequate affinity. The second selection involves a positive stimulation to select cells that can interact with major histocompatibility complex (MHC) molecules. MHC-I is present on nucleated cells to allow antigen presentation to CD8^+^ T cells while MHC-II is expressed on antigen-presenting cells and allows the presentation to CD4^+^ T cells [12]. The final selection or central tolerance removes self-reacting thymocytes by apoptosis. However, some self-reactive T cells still enter the bloodstream. To counteract this, natural regulatory T cells (nTreg), constituting ~10% of human CD4^+^ thymocytes [13] arise concurrently with naïve T cells (TN) to induce peripheral tolerance by (i) inhibiting the activation of self-reactive T cells and (ii) secreting anti-inflammatory cytokines to shut down T cell-mediated immunity at the end of an immune response to prevent autoimmunity. Thymic selection eliminates more than 90% of thymocytes [9]. The remaining cells exit the thymus as TN and circulate between blood and secondary lymphoid organs (SLO) (lymph nodes, spleen and tonsils) where they await activation through the presentation of specific antigens.

Thymic production of TN cells declines within the first 2 years of human life, is very low up to 15 years of age and by 35–40 years of life, there is minimal thymic output [10]. T cells are primarily involved in tolerance, pathogen responses and immunological memory formation in childhood, while in adulthood they control infection, maintain homeostasis, regulate the immune system and maintain pathogen and auto-antigen surveillance. There have been two prevailing theories regarding the ontogeny of memory cells, but neither has yet been proven: the progressive differentiation model [14,15,16], and the asymmetric division model [17].

**Figure 1:**
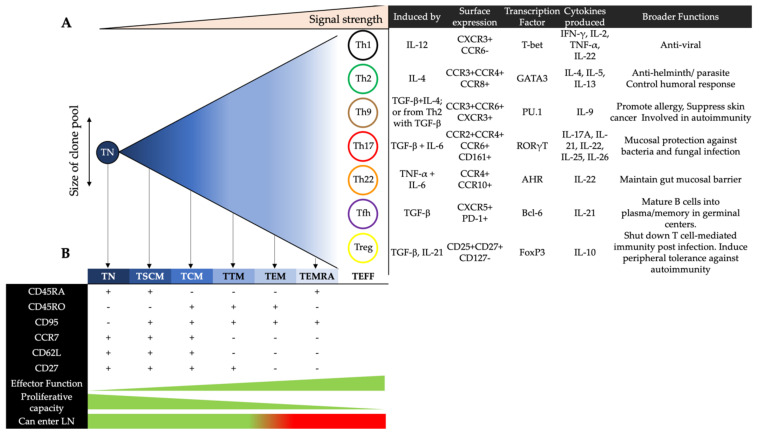
The linear model of progressive differentiation. (**A**) The differentiation of a naïve T cells (TN) cell into an activated phenotype that ultimately results in cell death. Once a CD4^+^ T cell reaches its full differentiation potential, and depending on stimuli, it can be defined as one of seven major effector helper subsets, with distinct surface markers, cytokine secretion profiles and overall functions. (**B**) As a cell progresses down this linear path, it may diverge at any point depending on the strength and consistency of the stimulus. This divergence results in a memory cell phenotype depending on the early level of differentiation the cell has already experienced. These memory cells are defined by the expression of a range of surface markers and are distinguished by the strength of effector functions and proliferative capacities. According to this model, once a memory cell is reactivated, the cell must only travel down the linear progression to become a short-lived effector T cell (TEFF). This process cannot be reversed to an earlier subset.

The progressive differentiation model suggests that there is a linear progression of memory cell differentiation, starting from TN, to stem cell memory (TSCM), central memory (TCM), transitional memory (TTM), effector memory (TEM), terminally differentiated effector memory (TEMRA) T cells and culminating in a large pool of short-lived effector T cells (Teff). How far the cells progress down this scale is determined by the consistency and strength of the inflammatory signal during the activation process. As cells follow this progression, they lose the ability to proliferate, but respond better and more quickly to pathogens via the release of effector cytokines [14,15,16] (Figure 1). The “asymmetric division model” relies on the idea that a cell’s “fate” is determined early, whereby any two daughter cells are not identical; one is committed to a memory cell and the other is committed to an effector cell [17]. Effector cells can be divided into seven major subsets, delineated by their surface protein expression, transcription factors and functional response [18]. However, their relationship with memory cells remains largely unclear in this model.

### 2.2. Effector CD4^+^ T Cells in Blood

Th1 cells primarily secrete IFN-γ to promote adaptive responses against intracellular pathogens [19]. They can also produce IL-2, IL-22 and TNF-α [20,21]. Th2 cells express the transcription factor GATA-3 and secrete IL-4, IL-5 and IL-13. They maintain immunity against helminthic parasites but also regulate humoral responses by driving B cell proliferation and IgE class-switching of antibodies [22]. In contrast, Th9 cells express PU.1 and produce IL-9. They are implicated in allergic inflammation [23], tumor suppression [24], and autoimmune disease [25]. Th17 cells secrete IL-17, which promotes the expression of antimicrobial agents by epithelial cells to protect against mucosal pathogens [26]. Of the six IL-17 proteins (A-F), IL-17A defines the Th17 subset [27]. Th17 cells can also produce IL-21, IL-22 and IL-26 [26,28]. IL-22 maintains the structural integrity of the gut epithelial barrier and protects against mucosal pathogens by stimulating enterocyte production of antimicrobials [29]. As Th17 and Th22 cells both secrete IL-22, they share similar functions in preserving gut homeostasis [30]. Whilst both subsets express CCR5 and CCR6, Th22 cells are phenotypically distinguished from Th17 cells as they express CCR10 and AHR [20,30,31]. Regulatory T cells (Treg) modulate inflammation through the release of immunosuppressive cytokines (IL-10 and TGF-β), consumption of IL-2, and therefore apoptosis of other T cells [32]. Tregs express CD25, CD27 and FoxP3, but lack CD127 [32,33,34]. Follicular helper T cells (Tfh) express CXCR5 and reside in the germinal centers of lymphoid follicles. They are involved in humoral immunity as they promote the class-switching and somatic hypermutation of antibodies, as well as the development of memory B cells [35]. Follicular regulatory T cells (Tfr) share transcription patterns with Treg and Tfh cells. They localise to the germinal centers of lymphoid follicles to inhibit the production of autoreactive antibodies by B cells [36] and to limit the frequency and activity of Tfh cells [37,38].

### 2.3. Memory CD4^+^ T Cells in Blood

Effector CD4^+^ T cells mediate immune responses that support the clearance of pathogens. Although many of these cells undergo apoptosis upon the resolution of infection, a small fraction differentiates into resting memory T cells to confer lasting immunity by promoting secondary responses during subsequent exposures [39]. It is currently unclear whether all the effector CD4^+^ T cell subsets can differentiate into memory T cells. There are five subsets of memory T cells that differ in their proliferative and survival capacities: TSCM, TCM, TTM, TEM, and TEMRA. As with the effector CD4^+^ T cell subsets, the memory CD4^+^ T cell subsets are also characterised by varying expression patterns of chemokine receptors that facilitate their homing from the circulation towards peripheral sites [40].

TSCM cells constitute between 2–4% of all circulating T cells and are the least differentiated subset, acting as the precursors of TCM cells [41,42]. They have enhanced longevity and self-renewal capacities compared to the other memory T cell subsets [14]. TCM cells traffic between the blood and SLO. Upon antigenic stimulation, these cells sequentially differentiate into TTM cells and TEM cells to exert effector functions [39,43]. TEMRA cells are terminally differentiated effector memory CD4^+^ T cells that re-express CD45^+^RA. Based on GPR56 expression, GPR56^-^ TEMRA cells are transcriptionally similar to TEM cells and have similar frequencies in the blood across individuals. In contrast, the frequency of the GPR56^+^ subset varies considerably, and these cells display cytotoxic features such as the expression of perforin and granzyme B [44].

### 2.4. The Paradigm Shift in T Cell Biology from Blood to Tissue

Over 20 years ago, two circulating memory populations of T cells were defined: TCMs have high proliferative capacity and express CCR7^hi^ allowing access to lymph nodes; TEMs have rapid cytotoxic response and are CCR7^lo^. Advanced technologies expanded the memory repertoire of T cell subsets (as discussed above [45,46]), and a shift in focus from blood and lymph nodes to peripheral tissue sites revealed the existence of T cells that reside in tissues long after pathogen clearance [47] to provide long-term protective immunity. All tissues contain a large repertoire of resident memory T cells, which increases in numbers with age [3,48]. This transition occurs during childhood in mucosal tissues (lungs and intestines) and in adulthood in lymphoid sites, with lymph nodes exhibiting the slowest transition to memory T cells [10]. How a TRM fits within the models of memory differentiation mentioned above is currently unknown. Two recent studies demonstrated the ability of mouse CD8^+^ TRM to leave tissue and join the circulating T cell pool as TCM or TEM following reinfection with influenza or lymphocytic choriomeningitis virus (LCMV), respectively [49,50]. Interestingly, at the transcriptional level, TRM were closer to TCM than TEM, thus providing a new “terrace model” of differentiation [51]. The terrace model maintains the major points of the progressive differentiation model but allows a cell to move between residency and circulation at any stage of its differentiation. In humans, Cutaneous Lymphocyte-associated Antigen (CLA)^+^CD103^+^ cells that downregulate the expression of CD69 were found to exit the skin through draining lymphatics and enter the blood of healthy donors. In a skin graft model, the CLA^+^CD103^+^ cells would re-seed in the engineered human skin, but not the surrounding murine skin. In contrast to the CD8^+^ studies, these cells did not require a re-stimulation in order to reseed original tissue sites. These apparent contradictions may derive from a difference in the migration processes of CD4^+^ versus CD8^+^ TRMs, or between mice versus humans. The terrace model finds a place for “residency” within the existing models of T cell differentiation. It captures a relationship between residency and circulation, suggests a role for TRMs in infection and reinfection events and does not wholly diminish the models that came before it.

## 3. Human CD4^+^ Tissue Resident Memory T Cells

### 3.1. Tissue Retention and Homing Markers on CD4^+^ TRMs in Different Anatomical Sites

A CD4^+^ TRM cell is defined by its static presence long after pathogen clearance, the expression of tissue retention marker CD69 and/or CD103 (much less expression than for CD8^+^ TRMs), upregulation of the survival and homeostasis marker CD127 [52] and a decrease in markers that promote egress such as KLF2, CCR7, S1PR1 and CD62L [1,53]. The Programmed cell death protein 1 (PD1), which is known to have an inhibitory role in the process of T cell activation [54,55,56] is enriched on CD69^+^ T cells isolated from tissues [1,57,58,59]. In addition, the upregulation or downregulation of certain markers such as CLA and CCR9 allows homing to specific tissues [1,39] such as skin and colon, respectively [2]. While CD8^+^ TRMs are more transcriptionally homogenous, the phenotypes of CD4^+^ TRM cells are more heterogenous in different human tissue. In human skin, CD4^+^CD69^+^ TRM cells have higher CD103 expression in epidermis compared to dermis [60], in contrast to TRMs in bowel expressing low CD103 [2,3]. This difference is derived from the local tissue environment: the skin is rich in TGF-β and interactions of T cells with keratinocytes drive CD103 expression in a TGF-β-dependent manner [60]. In human gastrointestinal tissue (GIT), CD8^+^ CD69^+^ T cells are either CD103 high or low with distinct functions [61] while CD4^+^ CD69^+^ T cells do not express CD103 [2,3]. In human genital mucosa, CD103 expression was found primarily in epithelium and on CD8^+^ T cells [62]. CXCR6 was detected on T cells derived from lung, spleen colon, skin, lung and liver [1,2] and contributes to CD8^+^ TRM development by directing T cell homing [63,64,65,66]. CXCR3 is upregulated on brain-derived CD69^+^ CD4^+^ T cells [67]. The identification of CD4^+^ TRMs with specific markers is challenging as their phenotype may vary according to the tissue type.

In addition, the method of CD4^+^ T cell isolation may influence the phenotype of these cells [68]. For example, in cervicovaginal lavage samples, a substantial portion of migratory cells was identified as CCR7^+^CD69^+^ cells [69], while the enzymatic digestion of cervical tissue explants yielded cells with the phenotype CCR7^–^CD69^+^ [57], suggesting that CCR7^+^ cells are absent in cervix, or that CCR7 detection was hampered by enzymatic digestion. Cervicovaginal lavage does not represent the total TRM populations within the cervix, while the latter study used a more conventional method to isolate all TRMs. Similarly, when bronchial lavage and enzymatically digested lung tissue were compared, different proportions of CD69 and CD103 expressing cells were found in both the CD4^+^ and CD8^+^ T cell subsets. Furthermore, the proportions of CD4^+^ cells in lavage samples were significantly lower than those isolated from airway and lung tissues [59]. Thus, the isolation methods used to study CD4^+^ TRMs [1,2,3,70,71] should be carefully considered in addition to the type of human tissues and whether the tissue source is healthy, inflamed or otherwise diseased.

### 3.2. Ontogeny of CD4^+^ and CD8^+^ TRMs in Mice and Humans

In mice, CD69 expression combined with the downregulation of KLF2 and S1PR1 establish CD8^+^ T cell tissue residency [72]. To reveal potential early determinants for TRM cell residency, single-cell RNA techniques were used to track the differentiation of CD8^+^ T cells in mice during the course of LCMV infection [73]. Findings suggested that the local microenvironment dictates transcriptional changes that occur after a T cell reaches the tissue and that the early determinants may differ between tissues and exposure to different pathogens. However, there is little direct evidence for what drives the establishment of human TRMs, much less CD4^+^ T cells. A recent study assessed TRM formation in human lung tissue transplanted from an HLA-discordant donor [59]. Through single-cell RNA analysis of mostly CD8^+^ T cells, TRMs identified in the recipient up-regulated expression of Recombination Signal Binding Protein for Immunoglobulin Kappa J Region (RBPJ), supporting the idea of Notch signalling pathways’ involvement in human TRM ontogeny [58]. Notch signatures have been identified in both CD4^+^ and CD8^+^ TRMs in humans, but only in CD8^+^ TRMs in mice. In addition, other studies have shown that TRM express transcriptional factors implicated in CD8^+^ TRM ontogeny in mice: Hobit, Blimp-1, [74] and RUNX3 [75]. In summary, our current understanding of human TRM ontogeny is in its preliminary stages, as experimental approaches such as parabiosis (by which the blood circulation of two individuals are conjoined) cannot ethically be carried out in humans. Furthermore, both disease state and tissue type can influence conclusions [73] and tissue-specific studies are needed to reveal the ontogeny of CD4^+^ TRMs in genital mucosa.

### 3.3. Tissue Entry and Exit

Before a cell enters a tissue, it undergoes a process of adhesion and rolling along endothelial surfaces in supplying capillaries [76]. This requires the expression of E- and P-selectin ligands on T cells [77]. Signals that arrest the rolling steps to allow migration differ between tissue types, healthy and inflamed status. Endothelial cells in the colon constitutively express CCL28 [78] and MAdCAM-1, which arrest cells expressing CCR10 and the gut-homing receptor α4β7, respectively [79,80]. Endothelial cells in the skin constitutively express CCL17 and CCL27, which arrest cells expressing CCR4 [81] and CCR10 [82], respectively. In inflammation, expression of CCL20 and CCL8 in inflamed skin arrest cells expressing CCR6 and CCR8, respectively [83,84]. In the context of HSV infection and the female genital mucosa, CXCL9 drives the recruitment of T cells expressing CXCR3 [85,86,87]. Lastly, the cell needs to adhere to and migrate across the endothelium. This is mediated by LFA1-ICAM1 and homophilic platelet/endothelial cell adhesion molecule (PECAM-PECAM) interactions, respectively [88]. The cell is then exposed to the signals that mediate maintenance. In mouse skin, CD103^+^ CD8^+^ T cells are a slow-moving population located in the epidermis whereas CD4^+^ T cells are more ubiquitously distributed throughout tissues due to their motility and migratory function [77,89]. Integrins induce motility as in mouse lung following infection with influenza; CD49a^+^ (integrin α1) CD8^+^ T cells exhibited higher motility than their static CD103^+^ (integrin αE) CD8^+^ T cell counterparts [90]. However, CD103 is expressed on egressing CD4^+^ T cells in mice and human [91,92]. CLA^+^CD103^+^CD4^+^ T cells in draining lymph nodes of human skin (and in blood) are transcriptionally similar to those in tissues, raising doubts whether CD4^+^ T cells do maintain prolonged residency. However, conjoining the blood circulations of two mice (parabiosis) demonstrated that CD4^+^ TRMs are distinct from the recirculating counterparts [93] and their gene expression signatures are specific to different tissues, lineages and migration capacities, broadening the heterogeneity of murine CD4^+^ TRMs as mentioned above. Even though donor CD4^+^ T cells were found infrequently in lung tissue after transplantation, and long-term mature CD8^+^ TRMs predominated [59], these studies in mice indicate that CD4^+^ T cells can remain resident to provide protection for prolonged periods of time.

### 3.4. Maintenance of CD4^+^ and CD8^+^ TRMs Via Cytokines and Cell Interactions

Cytokines: Little is known about the contributions of cytokines towards the development and maintenance of TRMs. IL-15 produced by keratinocytes, intestinal epithelium and monocytes [94], and IL-7 produced by human intestinal epithelium [95] are pro-survival and support homeostatic proliferation [96,97]. In humans, the IL-7 receptor (CD127) is expressed on CD4^+^ TRMs derived from GIT, lung, skin, colon, and cervix [2,48,57,71] while two of the heterotrimeric receptor chains for IL-2 and IL-15 [CD122 (β chain) and CD132 (γ chain)] are enriched on CD4^+^ TRMs in cervical tissue [57]. In human bone marrow, CD69^+^ T cells are associated closely with IL-15 producing cells [98].

In mouse skin and GIT, both IL-7 and IL-15 maintain CD8^+^ TRM cells [48,99] while IL-7 alone [100,101] or in combination with IL-15 [102] maintains CD4^+^ TRMs. A combination of IL-2, IL-15 and TGF-β induced CD8^+^ murine ex-TRMs (cells that have re-entered circulation) to revert to a TRM phenotype as measured by the upregulation of CCR9 and CD69 [49]. TGF-β regulates CD103 on CD8^+^ T cells to induce a long-lived TRM phenotype or to immobilise them [60,103]. CD103-eCadherin interaction binds TRMs to keratinocytes [104]. However, in mice, CD103^−^CD8^+^ TRM can also develop without TGF-β in intestinal lamina propria [105], whereas CD103 induced by TGF-β is required for TRM formation in the intestinal epithelium [106]. CD69 expression in CD8^+^ TRM cells in mice does not rely solely on TGF-β and requires IL-33, IFN-α/β, TNF-α [106] and IL-12 [107], but not IFN-γ.

**Figure 2:**
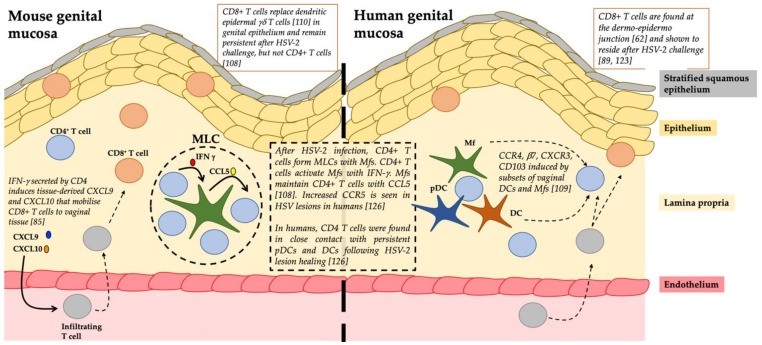
A summary of the role of resident memory T cells in the mouse and human genital mucosa especially during herpes simplex virus (HSV) infection.

Cell Interactions: Following HSV-2 challenge in the genital tract of mice, CD4^+^ T cells form memory lymphocyte clusters with Mfs; IFN-γ produced from the CD4^+^ T cells activate Mfs, to produce CCL5 that maintains CD4^+^ T cells (Figure 2) [108]. A study of DC subsets in human vaginal mucosa demonstrated that these subsets can induce tissue retention markers on CD4^+^ T cells [109]. In mice, CD8^+^ T cells are known to compete with, and over time, replace dendritic epidermal γδ-T cells within the epidermal niche of skin [110]. CD4^+^ helper T cells play a key role in priming cytotoxic CD8^+^ T lymphocyte (CTL) responses and in promoting memory CD8^+^ T cell development [111]. While migration of CD8^+^ T cells into mucosal tissues of the lung and intestine does not require CD4^+^ help or inflammation [112], other peripheral mucosal sites such as the vagina rely on CD4^+^ to mobilise and control CD8^+^ migration from blood to the infected tissues via secretion of IFN-γ and the inflammatory chemokines, CXCL9 and CXCL10 *in situ* (Figure 2) [85]. It has also recently been suggested that virus-specific CD4^+^ TRMs help develop anti-viral CD8^+^ TRMs through IL-21 secretion [4,113].

## 4. The Role of TRMs in HSV Infection

HSV type 1 (HSV-1) predominantly causes oral, ocular and initial genital herpes while HSV-2 causes initial and recurrent genital herpes. An estimated 16–17.6% of the world’s population aged 15–49 years of age (596–655.7 million people) have genital HSV-1 and/or HSV-2 [114]. No vaccine is currently available, and the correlates of protection are only partly established [115]. The importance of both CD4^+^ and CD8^+^ T cells in response to HSV infection has been studied over many years and is known to operate at two sites: the neuronal ganglia and the skin/genital mucosa. At the neuronal ganglia, CD4^+^ and CD8^+^ T cells surround the neurons where they control latency and suppress reactivation [116,117]. In human trigeminal ganglia, IFN-γ^+^ TNF-α^+^ CD4^+^ and CD8^+^ T cells have been observed in clusters around neurons and recognise distinct HSV-1 epitopes [118]. However, whether these T cells were truly TRM cells was not determined.

HSV-1 and 2 invade the stratified squamous epithelium of the mucosa lining the anogenital tracts. This epithelium consists of 7–10 layers of keratinocytes, a network of interconnected Langerhans cells (LCs) and the newly described type 2 DCs (cDC2s). In human recurrent genital herpes lesions, CD4^+^ T cells infiltrate the dermis and lower epidermis first and predominate in the first 12–48 h [119]. They produce IFN-γ to combat HSV immune-evasive mechanisms in infected keratinocytes [120] and to stimulate epithelial secretion of CXCL9 and CXCL10 for the recruitment of CD8^+^ T cells to the infection site [85]. The subsequent infiltration of CD8^+^ T cells is strongly correlated with viral clearance [121].

In the human female genital tract, HSV-2 specific CD8^+^ TRMs persist at the dermo-epidermal junction after lesion healing, surveying the adjacent peripheral nerve endings for HSV shedding [122,123]. However, studies into the localisation and density of CD8^+^ TRMs have revealed that there is spatial heterogeneity and that these cells are static and thus reliant on cytokines such as IFN-γ for the majority of their antiviral effect rather than cytotoxicity. Thus, HSV-2 can exploits gaps between the cytokine influence of these cells, enabling some viral shedding to occur at these gaps. [124,125].

The role of CD4^+^ TRMs in HSV infection has not been as well studied as that of CD8^+^ TRMs, and thus their role is less clear. In mice it was shown that migrating and resident memory T cells intersperse to establish long-term memory against HSV-1; however, key differences in the localisation of memory CD4^+^ and CD8^+^ T cells occurred following infection. CD8^+^ memory T cells established a static, resident population in the epidermis at the original site of infection, while a dynamic population of CD4^+^ T cells trafficked through the dermis and re-entered the circulation [77]. However, the authors could not rule out the existence of resident CD4^+^ T cells that remain in the dermis long-term.

Iijima and Iwasaki investigated whether they could establish a CD4^+^ TRM population in the vagina of parabiotic mice that were immunised intravaginally with an attenuated strain of HSV-2 [108]. CD4^+^ TRMs were identified in memory lymphocyte clusters (MLCs) together with Mfs and DCs in vaginal submucosa and the surrounding hair follicles in the upper dermis of skin were the predominant site of CD4^+^ TRM cells. Upon HSV-2 re-infection, mice relying on only circulating CD4^+^ memory T cells could not fully suppress viral replication, whereas mice harbouring HSV-2 specific CD4^+^ TRMs were fully protected. Therefore, the establishment of CD4^+^ TRMs was critical for complete protection from disease. Furthermore, it was determined that CCL5 was upregulated in vaginal tissue following immunisation and that CCL5 was required to retain CD4^+^ TRMs at the site of infection [108]. A subsequent mouse study found that CD4^+^ TRMs were found in the dermis with most clustered in MLCs and around hair follicles after HSV infection. CCL5 secreted by Mfs was critical in retaining the CD4^+^ TRMs in these clusters (Figure 2) [76,91]. CD4^+^ TRMs were mobile, much more than CD8^+^ TRMs and able to migrate to distal areas of the skin in response to pathogens. These MLCs containing CD4^+^ TRMs may be an “immediate response centre” able to respond quickly to incoming pathogens. In contrast, circulating CD4^+^ TEMs would need to undergo all the phases of entry (rolling adhesion and migration) and proliferation to combat pathogens [76].

In human studies, an initial study in biopsies from recurrent herpes lesions showed that CD4^+^ T cells along with CD8^+^ T cells persisted at the site of reactivation for months after lesion healing [126] indicating a protective role of persisting CD4^+^ T cells in localised tissue. Similar to the murine studies, the authors found these populations localised in the upper dermis long after HSV-2 clearance, and also showed them interacting with dermal DCs [126]. In contrast, the resident memory CD8^+^ T cells were more superficial at the dermo–epidermal junction. Enriched HSV-2 specificity of these persisting CD4^+^ T cells in areas of prior HSV-2 reactivation [126] highlights the important role of CD4^+^ TRMs in protection against HSV. The various myeloid and lymphoid cell types and their relationships in the human dermal clusters require further definition. Recently, a mathematical model of the spatial dispersions of CD4^+^ and CD8^+^ TRMs in the human genital tract showed that in their absence, HSV-2 infection will continue to expand [127]. Furthermore, cytokines derived from a low density of HSV-2 specific CD4^+^ TRMs can rapidly diffuse to contain the spread of HSV-2 infection, thus highlighting the impact of CD4^+^ TRMs in eliminating HSV-2 infected cells [127].

### 4.1. Herpes Keratitis

More than 60% of new HSV-1 infections in the eye are epithelial keratitis [128,129,130] where HSV has infected the outermost layer of the cornea, the epithelium [131]. Herpes keratitis (HK) generally resolves on its own although antiviral therapy is often utilised to speed recovery and prevent damage to the cornea [132,133]. The stroma is the thickest layer in the cornea and consists of organised collagen fibres [134] allowing transparency in the cornea which is essential for vision [135]. With recurrent episodes of HSV infection in the stroma, herpes stromal keratitis can lead to corneal opacification and can result in permanent vision loss [136]. A major complication from HSV-1 infection is ocular herpes, especially HK, resulting from reactivation of latent HSV infection in the trigeminal ganglion [137]. Physiological, environmental factors, and chemical stresses [131] allow the virus to travel via anterograde axonal transport [138] to the surface of the cornea [133,139,140]. HK is currently the leading cause of infectious blindness in developed countries [129,131] and affects 1.5 million people globally each year [128,130,137,141,142]. Thus, there is a global need to develop a vaccine for HSV-1 and -2.

### 4.2. Early Response to Herpes Keratitis Involves the Innate Immune System

The host response to HSV infection in the eye is complicated and remains to be fully defined. Whilst there are many gaps in understanding the innate immune response to HK in humans, there has been much research in murine models of HK. Studies in mice have established that in response to HSV-1 infection, neutrophils are first to arrive at the site of infection [143,144]. They arrive in two phases in a large influx and are believed to play an important role in clearing HSV from the cornea [145,146,147]. However, the second phase has also been shown to cause significant tissue damage and opacity in the cornea [148]. As the cornea is an avascular tissue, it was previously thought that the cornea was immune-privileged [149]. However, studies in normal murine corneas show that some immune cells of the innate immune system are present in this tissue [150,151,152]. Upon HSV-1 infection, conventional DCs (cDCs) and Mfs are activated and increase in numbers [153,154,155,156,157]. The depth of insight regarding the innate immune system in human tissue is limited. The depletion of cDCs in mice has shown increased scarring whilst the depletion of Mfs had no impact, suggesting that these cDCs are critical for maintaining corneal health during HSV infection [158]. Additionally, the depletion of natural killer cells in other mice studies showed that the incidence and severity of scarring in the stroma were significantly reduced [159,160]. These studies indicate that natural killer cells are activated upon HSV infection and cause damage to the cornea in order to clear the virus. Ex vivo HSV infection of normal human corneas showed the co-localisation of DCs, LCs and Mfs with HSV infected corneal cells [161,162]. The localisation of these antigen-presenting cells in human tissue confirms similar studies in mice, suggesting that antigen-presenting cells are involved in early immunopathology of HSV infection in the cornea.

### 4.3. Late Stage Response to Herpes Keratitis Involves T Cells

#### 4.3.1. CD4^+^ T Cells

The later stages of HK display the presence of T cells that are particularly prominent in driving inflammation in the stroma [163,164]. Mice studies show that upon HSV-1 infection, CD4^+^ T cells appear in the cornea and continue to increase in numbers with time. Furthermore, the depletion of T cells showed that mice were less susceptible to tissue damage in the cornea upon HSV-1 infection. In the literature, the subset of CD4^+^ T cells, specifically Th1 cells that produce IL-2 and IFN-γ, facilitate the influx of second wave neutrophils [145,165,166]. Thus, Th1 cells are considered responsible for the inflammation and scarring seen throughout the stroma. Another subset of CD4^+^ T cells, Th17 cells which produce IL-17, also contribute to immunopathology as mice studies examining ocular HSV infection found reduced scarring in IL-17R knock-out mice [167]. Furthermore, in a donor cornea from a patient diagnosed with HK, IL-17 was expressed implicating Th17 cells in the pathogenesis of HK [168]. Similar studies utilizing T cell lines revealed the presence of HSV-specific CD4^+^ T cells in corneas of HK patients [169,170]. These cells secreted IFN-γ, IL-2, Il-4, and IL-5 upon HSV-specific stimulation suggesting the critical role CD4^+^ T cells have in the immunopathology of HK in humans. In contrast, CD4^+^ Tregs limit scarring as scarring in the stroma was greater in mice that were Treg depleted before HSV infection [171,172,173]. Additionally, if Tregs were transferred adoptively to mice, corneas showed less scarring compared to controls [171,172], suggesting that Tregs are beneficial to corneal health during HK.

#### 4.3.2. CD8^+^ T Cells

At the level of the trigeminal ganglion, several groups have shown infiltration of CD8^+^ T cells after primary infection in both humans and mice, and have demonstrated that in mice these CD8^+^ T cells synergise with CD4^+^ T cells to control HSV-1 reactivation. Thus HSV-1 specific CD8^+^ T cells can migrate into the trigeminal ganglia and retain memory phenotype presumably as CD8^+^ TRMs, although this requires definitive proof in humans [174,175]. More recently another group, using a virulent strain of HSV-1, has produced evidence that it is CD8^+^ DCs that control such reactivation and that CD8^+^ T cells are merely bystanders [176]. These contradictions are yet to be fully resolved. Other studies investigated subpopulations of CD8^+^ T cells. Polyfunctional effector memory CD8^+^ T cells were found in increased proportions in asymptomatic humans seropositive to HSV, in comparison to symptomatic patients with a history of recurrent HK and monofunctional CD8^+^ T cells [177]. Furthermore, immunisation with MHC-I restricted epitopes in adenoviral vectors in mice, induced together with the T cell chemokine CXCL10, elicited a strong CD8^+^ T cell-dependent protective immunity against HK [177], suggesting the importance of inducing CD8^+^ T cells in vaccine development [178]. In contrast, another group has shown evidence for induction of corneal scarring, probably mediated by HSV glycoprotein K stimulated CD8^+^ T cells [179]. Thus, protection or immunopathology may depend on different HSV antigens and requires further study. In summary, in primary murine HK CD4^+^ T cells play a major immunopathologic role, and CD8^+^ T cells appear to offer both protection and immunopathology in different settings and are yet to be fully defined. However, the role of CD4^+^ and CD8^+^ TRMs in the control or pathogenesis of recurrent HK at the level of the trigeminal ganglion or cornea is yet to be defined and technically very challenging to study.

## 5. The Role of CD4^+^ T Cells in HIV Infection

**Figure 3:**
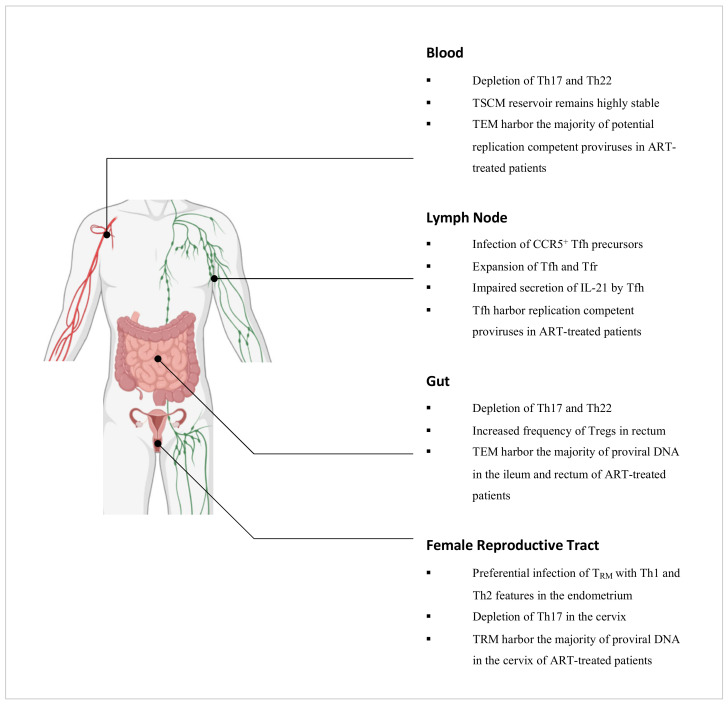
Summary of the key dynamics amongst CD4^+^ T cell subsets during HIV infection in relevant anatomical compartments. Figure created with BioRender.com.

It is well established that the natural course of untreated HIV infection is characterised by persistent HIV replication and a progressive decline in CD4^+^ T cells which results in the onset of immunodeficiency [180]. Antiretroviral therapy (ART) can inhibit viral replication such that the viral load is reduced to clinically undetectable levels. It is however not curative owing to the presence of HIV that remains in a quiescent state in CD4^+^ T cells which are unaffected by the immune system or ART [181]. Errors during reverse transcription give rise to defective proviruses that constitute more than 93% of latent proviruses in HIV-infected individuals on ART [182,183,184]. Though the prevalence of replication-competent proviruses is low, they persist nonetheless as the cessation of sustained ART results in a rapid rebound of viral load [185]. The latent reservoir is therefore a key impediment towards the elimination of HIV in infected individuals. In this section, we review the roles of effector and memory CD4^+^ T cell subsets in relation to HIV infection and persistence (Figure 3).

### 5.1. Effector CD4^+^ T Cell Subsets and HIV Infection

#### 5.1.1. Th1 Cells

In a recent study by Orlova-Fink et al., Th1 cells were found to express high levels of CCR5, correlating with in vitro infection assays which showed that these cells were susceptible to infection by R5-tropic HIV, more so than with the X4-tropic counterpart [186]. In another study by Lee et al., genetically intact and potentially replication-competent HIV proviral DNA was found to be enriched within Th1-polarised CD4^+^ T cells, compared to other functionally polarised CD4^+^ T cell subsets derived from the peripheral blood of HIV-infected individuals on ART [187]. Such Th1-polarised cells were further found to have undergone clonal expansion, implicating them as a driving force towards the maintenance of the latent reservoir [187].

#### 5.1.2. Th2 and Th9 Cells

Limited studies have interrogated Th2 and Th9 cells in the context of HIV. It is however known that these cells are more permissive to infection with X4-tropic HIV than the R5-tropic strain in vitro, correlating with their high surface expression of CXCR4 but not CCR5 [186,188]. X4-viral sequences have further been reported to be preferentially detected in Th2 and Th9 cells derived from the peripheral blood of HIV-infected individuals [186].

#### 5.1.3. Th17 Cells

Th17 cells are highly permissive to HIV infection, in part due to their heightened expression of HIV receptors, but also their concurrent lack of autocrine CCR5 ligands and RNase proteins which can inhibit viral replication [189,190]. In simian immunodeficiency virus (SIV)-infected macaques and HIV-infected individuals, Th17 cells have been reported to be depleted in the peripheral blood and the intestinal mucosa [30,191,192,193]. The depletion of such cells has been associated with functional perturbations of the intestinal epithelial barrier and is thought to promote microbial translocation as well as chronic inflammation [194]. Importantly, sustained virologic suppression by ART does not restore the Th17 cell population in the gut in the majority of HIV-infected individuals [195]. Although these cells typically home towards the gut via the CCR6-CCL20 chemotactic axis, this is impaired in ART-suppressed, HIV-infected individuals as there is a decreased production of CCL20 by enterocytes in response to IFN-γ secretion by Th1 cells as well as IL-10 and TGF-β secretion by Tregs [196,197]. Recently, Th17 cells were found to be enriched in the inner foreskin of uninfected men compared to the outer foreskin [198]. The dynamics of these cells in this tissue during HIV infection however are yet to be established. By contrast in the female reproductive tract, Th17 cells have been implicated as preferential targets for infection during the vaginal transmission of SIV in macaques [199], and have further been found to be depleted in the cervix of HIV-infected female sex workers [200,201].

#### 5.1.4. Th22 Cells

In SIV-infected macaques, depletion of Th22 cells has been observed in the peripheral blood as well as the colon and rectum [30,191]. Such findings are concordant with studies of HIV-infected individuals which have also reported reduced frequencies of Th22 cells in these compartments [202,203,204], and this has been associated with damage towards the mucosal barrier and increased microbial translocation [204]. Although virologic suppression by ART does not restore the frequency of Th17 cells in the gut due to perturbations in the CCR6-CCL20 chemotactic axis [195,196,197], Th22 cells, by contrast, are reconstituted [195]. These cells preferentially home towards the gut in a CCL20-dependent manner but can migrate via the CCR10-CCL28 chemotactic axis as an alternative when CCL20 levels are reduced as in ART-suppressed HIV-infected individuals. It is important to note that despite the restoration of the gut Th22 cell population following ART intervention, these cells are unable to functionally compensate for the depletion of Th17 cells that persists [195]. Recently, Th22 cells were found to be enriched in the outer foreskin of uninfected men compared to the inner foreskin [198]. The role of these cells in this tissue during HIV infection however remains to be explored.

#### 5.1.5. Regulatory T Cells

In a study by Shaw et al., HIV-infected non-controllers were found to have higher frequencies of Tregs in the rectal mucosa compared to HIV-infected controllers and uninfected individuals [205]. Mucosal Tregs across all cohorts were however found to limit the proliferation of autologous non-Tregs to similar extents [205], pointing towards the preservation of their suppressive capacities during chronic HIV infection and the potential contribution of Tregs towards diminished HIV-specific T cell responses [33]. Although ART intervention can reduce the frequency of Tregs in the rectal mucosa, it does not normalise to levels prior to infection [206]. Similarly in the cervix, Tregs have been reported to be increased in frequency during chronic HIV infection and this persists despite sustained ART [207]. In a study by Li et al., it was determined that Tregs could inhibit HIV replication in resting CD4^+^ T cells through a cAMP-dependent PKA pathway [208]. It has been postulated that during chronic HIV infection, this may contribute towards conditions that are conducive to HIV latency. Importantly, Tregs have also been implicated as a key cellular reservoir. In a separate study by McGary et al., CTLA-4^+^PD-1^–^CD4^+^ T cells, which are mostly composed of Tregs, were found to harbor proviral DNA outside the lymph node follicles in ART-treated, SIV-infected macaques [209]. Such findings were also observed in HIV-infected individuals on ART [209].

#### 5.1.6. Follicular Helper T Cells

Tfh cells are expanded in the lymph nodes of macaques and humans during SIV and HIV infection respectively [210,211], and accumulating studies have pointed towards the presence of proviral DNA in these cells suggesting their infection with SIV and HIV [212,213]. Although Tfh cells express CXCR4, these cells lack CCR5 expression and yet R5-viral sequences have been reported to be preferentially detected in Tfh cells [213]. Recent studies have identified that SIV and HIV can infect Tfh precursor cells which express CCR5 [214]. Upon TCR stimulation, this precursor population downregulates CCR5 expression and differentiates into Tfh cells [214], and this reflects a possible pathway by which Tfh cells may become infected with SIV and HIV. Importantly, though CTLs can clear infected cells in extra-follicular regions of lymph nodes [215], they have been found to be limited within the follicles [216]. Such findings, in combination with limited ART drug penetration in lymphoid tissues, implicate follicular sites as sanctuaries for the persistent replication of HIV [217]. In a study by Banga et al., Tfh cells derived from the lymph nodes of ART-suppressed, HIV-infected individuals were found to be a key source of replication-competent HIV proviruses [218], and so novel strategies to eliminate HIV will need to account for this unique cellular reservoir.

#### 5.1.7. Follicular Regulatory T Cells

These cells are highly permissive to R5-tropic HIV ex vivo, more so than Tfh cells and this is attributable to their elevated expression of CCR5 [219]. Using macaque models, several studies have reported significantly decreased ratios of Tfr cells to Tfh cells during chronic SIV infection compared to uninfection [220,221,222], negatively correlating with increased frequencies of autoreactive antibodies in the peripheral blood [220]. However, another study by Miles et al. found that the ratio of Tfr cells to Tfh cells was significantly increased during chronic SIV infection compared to uninfection [223]. In humans, Tfr cells were found to be more frequent in the lymph nodes in chronically HIV-infected individuals compared to in uninfected individuals [223]. By establishing an ex vivo human tonsil model of HIV infection, Miles et al. showed that Tfr cells were able to suppress the proliferation of Tfh cells as well as their production of IL-4 and IL-21 [223]. These cytokines support the maturation of high-affinity B cell clones and so their reduced secretion by Tfh cells may explain the impaired B cell responses that are displayed by HIV-infected individuals [224]. The disparity between studies nonetheless points towards the complexity regarding the role of the Tfr cell population in HIV infection and further studies are required to elucidate this.

### 5.2. Memory CD4^+^ T Cell Subsets and HIV Infection

#### 5.2.1. Stem Cell Memory T Cells

CD4^+^ TSCM cells are permissive to HIV infection, in part due to the decreased expression of HIV restriction factors such as APOBEC3G and SAMHD1 [225]. Whilst the contribution of these cells towards the latent reservoir is relatively low in the initial stages of ART, the CD4^+^ TSCM sub-reservoir remains highly stable over time as opposed to the other CD4^+^ memory T cell sub-reservoirs which progressively contract, and so the contribution of TSCM cells increases proportionately over long-term ART [226].

#### 5.2.2. Central, Transitional and Effector Memory T Cells

Of the memory CD4^+^ T cells, these subsets have been studied the most in relation to the latent HIV reservoir. In the peripheral blood of ART-suppressed HIV-infected individuals, two reservoirs have been identified which differ in their cellular compositions based on integrated HIV DNA. In individuals with restored CD4^+^ T cell counts, TCM cells have been found to harbor the majority of HIV proviral DNA. This first reservoir is maintained by antigen-driven proliferation and the intrinsically long-lived nature of TCM cells [227]. By contrast, TTM cells have been found to be highly enriched for HIV proviral DNA in individuals with low CD4^+^ T cell counts, and this second reservoir is thought to persist by means of homeostatic proliferation [227]. Importantly, integrated HIV DNA may however encompass defective proviruses [228], and a study by Hiener et al. found that TEM cells contained the majority of genetically intact and potentially replication-competent HIV proviruses in the peripheral blood [229]. In other anatomical compartments such as the ileum and rectum, HIV proviral DNA has been reported to persist mostly in TEM cells in ART-suppressed HIV-infected individuals [230], though whether these cells also contain the majority of replication-competent proviruses in these compartments is yet to be established.

#### 5.2.3. Terminally Differentiated Effector Memory T Cells

Although CD4^+^ TEMRA cells express high levels of CCR5, these cells are resistant to R5-tropic HIV infection presumably due to a block that occurs between viral entry and genomic integration. CD4^+^ TEMRA cells however remain susceptible to X4-tropic HIV infection [231]. In terms of the latent HIV reservoir, these cells only marginally contribute towards the pool of integrated HIV DNA in the blood [227].

#### 5.2.4. Tissue Resident Memory T Cells as key HIV target cells

Recent studies have pointed towards a role for CD4^+^ TRM cells as key targets for HIV infection and persistence, particularly in the female reproductive tract. In a study by Ma et al., CD4^+^ CD69^+^ TRM cells derived from the endometrium of uninfected women that displayed phenotypic features associated with Th1 (Tbet^+^) and Th2 (CRTh2^+^) cells were found to be a preferential target for HIV infection [232]. In a separate study by Cantero-Pérez et al., CD4^+^ CD69^+^ TRM cells derived from the cervix of uninfected women were found to be enriched for several proteins associated with susceptibility to HIV infection including α4β1, α4β7, CXCR4 and CXCR6. These cells were found to be preferentially infected by HIV compared to their cervical CD4^+^ CD69^–^ non-TRM counterparts, consistent with previous findings that cervical CD4^+^ T cells expressing α4β1, α4β7 or CD69 were preferential targets for HIV infection [233]. By comparing paired cervical tissue and peripheral blood samples derived from ART-suppressed HIV-infected women, Cantero-Pérez et al. further found that up to 200-fold more viral DNA molecules were present per cell in cervical tissue compared to in blood. Importantly, CD4^+^ CD69^+^ TRM cells were identified as the primary contributors to this tissue reservoir [57]. It should be noted however that the menstrual cycle may influence the proportion of CD4^+^ CD69^+^ TRM cells in the female reproductive tract [234], and so by extension, it may also affect the susceptibility of women towards HIV infection. In the colon and lymph node, CD4^+^ CD69^+^ TRM cells have also been implicated as contributors towards the latent reservoir. By reactivating latently infected CD4^+^ T cells derived from ART-suppressed HIV-infected individuals and tracing them back to their state before activation, Neidleman et al. found that latently infected cells of the colon and lymph node expressed CD69, suggesting that these cells were of a TRM phenotype [235]. Such findings collectively identify CD4^+^ TRM cells as potential targets for novel therapeutics and merit further studies of this subset in other tissues associated with HIV infection.

## 6. HSV and HIV Coinfection

### 6.1. Epidemiology

Many studies and recent meta-analyses have demonstrated that prior infection with HSV-2 increases the sexual acquisition of HIV approximately 3-fold. If HSV-2 infection is recent this increases to up to ~ 5-fold, likely because when HSV-2 infection is newly acquired there is an increased severity of ulceration, inflammation and shedding over the next year [236,237]. African studies showed acquisition of HIV usually occurs in an inflamed genital tract with or without ulceration [238], and that HSV-2 is responsible for >50% of female HIV infections [239]. HIV is also shed through herpetic lesions [240].

### 6.2. Pathogenesis

The mechanism of HSV2-enhanced HIV transmission is likely multifactorial. HSV-2 genital ulcers disrupt the stratum corneum, facilitating HIV entry into the epidermis. Additionally, activated CD4^+^ T cells and Mfs, target cells for HIV, infiltrate the upper dermis of herpes lesions, and remain between episodes, producing a persistent state of increased mucosal susceptibility [119,123].

Zhu et al. [126] examined HIV-1 replication in the dermal cellular infiltrate in sequential biopsies of HSV-2 lesions from patients with or without antiviral therapy. A mixed population of CD4^+^ and CD8^+^ T cells and myeloid DCs persisted at the sites of HSV-2 reactivation for months after healing and was unaffected by Aciclovir therapy. Suspensions of the lesional CD4^+^ T cells reacted to HSV-2 antigen and were enriched for expression of the chemokine receptor CCR5. Infection of these cells with an R5 strain of HIV showed higher concentrations of integrated HIV DNA in cells derived from healed genital lesion biopsies than in cells from control skin biopsies. Thus, the persistence and enrichment of CCR5^+^ CD4^+^ T cells in the genital mucosa provides further evidence for the ability of these cells to support increased HIV replication and spread. The lack of effect of anti-HSV-2 therapy on this replication is also consistent with the lack of effect of these agents in vivo to reduce HIV acquisition. Further characterisation of the CD4^+^ T cells as TRMs and the relative proportions of productive versus latently infected cells needs to be pursued.

Although the major HIV target cells, CD4^+^ T cells, are enriched in the dermis, HSV lesions are confined to the epidermis and yet enhance HIV acquisition. Coinfection or adjacent infection of epidermal LCs (or CD11c^+^ epi-cDC2s) may provide the conduit for HIV to reach dermal CD4^+^ T cells. Anogenital LCs are major target cells for infection by both viruses [241,242]. HIV and HSV have different and potentially converging interactions with DCs and T cells. HIV uses DCs for transfer to T cells where it replicates whereas HSV induces DC apoptosis [242,243,244] and uptake by bystander DCs for HSV antigen presentation to T cells.

Our laboratory studies of HSV transport within LCs to dermal DCs in clusters raise the question of whether HIV may be transported along the same route, either in coinfected or accompanying activated LCs or epi-cDC2s to interact with infiltrating T cells, perhaps within cell clusters [244]. Our lab has also previously shown that HSV infected LCs and monocyte-derived DC produce TNF-α which enhances expression of the HIV coreceptor CCR5, and therefore HIV infection of bystander LCs, supporting this hypothesis. TNF-α also enhances their migration [245]. In complementary studies, HSV-exposed DCs released cytokines that reactivated HIV from latency in U1 cell lines [246].

Furthermore, in SIV-macaque models, SIV has been shown to infect both activated and “resting” T cells in the dermis/lamina propria of the genital mucosa, leading to the spread of infected activated T cells to the lymph nodes. Infection of the resting T cells may initiate latent infection of these cells, the major impediment to antiviral eradication of HIV. Thus, it is possible that resting CD4^+^ TRMs in dermis between HSV recurrent lesions may be infected by HIV and become reactivated in the presence of pDCs and IFNα [247] or latent in the presence of myeloid DCs, as shown in model systems [248].

## 7. Vaccine Development

### 7.1. Herpes Keratitis

CD4^+^ TRMs are yet to be identified in the normal cornea and during HK. However, given the destructive nature of CD4^+^ T cells and the inconclusive role of CD8^+^ T cells during HK, further definition of CD4^+^ and CD8^+^ subpopulations are essential for vaccine development to HSV. As scarring from HK is a result of recurrent infection as opposed to primary ocular HSV infection, boosting the immune response via a vaccination may exacerbate the severity of scarring in the cornea [249,250,251]. Many vaccine trials typically exclude participants with a history of ocular HSV infection to prevent the potential exacerbation of corneal scarring [249]. However, as described earlier, success in producing immunity in mice with HSV epitopes inducing asymptomatic CD8^+^ T cells is encouraging for vaccine design against HSV [177]. Continued efforts to define T cell responses in HK are vital in propelling the future of vaccine design.

### 7.2. Genital Herpes

Systemic (intramuscular) injection of a vaccine candidate containing glycoprotein D and adjuvant MPL/Alum was partially successful (58–74%) in HSV seronegative women but insufficient for licensure [252,253]. This partial success remains a benchmark by which other candidate vaccines are measured. Numerous candidates have been developed since including specifically mutated live attenuated dl 5, 29 HSV-2, RNA vaccines and gD deleted HSV, with some in clinical trials [254]. Though CD4^+^ T cells have been induced and directed to the genital mucosa via vaccination, there has been no evidence to suggest these cells became resident.

Intradermal and mucosal applications of vaccine candidates have also been tested in animal models. Gebhardt et al. have suggested induction of CD8^+^ TRMs might be an important target for vaccine induction [255] and this principle was adopted in the “prime and pull” approach by Shin and Iwasaki [256]. This approach involves two steps: (1) priming of T cells by conventional systemic immunization and (2) recruitment of activated T cells to the genital mucosa by topical chemokine application. CD8^+^ T cells, but not CD4^+^ T cells, were retained in the long-term using this combined approach. HSV spread was reduced and clinical disease prevented. As genital herpes is thought to predispose up to 50% of HIV acquisition in sub-Saharan Africa, an HSV vaccine could potentially reduce HIV spread, and might prove to be more feasible than developing an HIV vaccine.

### 7.3. HIV

Currently, there is no effective vaccine against HIV. The modest success (31%) of the RV144 HIV vaccine [257] was not replicated in the HVTN702 clinical trial, and neither vaccine-elicited neutralizing Abs (nAbs) that maintained viral suppression in chronically infected HIV patients [258,259] or prevented infection in rhesus macaques infected with SIV [260,261]. Recently, macaques were immunized with an HIV envelope trimer which induced nAbs, and when combined with a viral vector, both nAbs and cellular immunity including CD8^+^ TRM T cells were elicited [262]. After ten viral vaginal challenges, protection was observed with both vaccines (53.3% for trimer and 66.7% for viral vector and trimer, respectively). A nAb titer >300 was required for protection with trimer alone but with viral vector and nAb, titers <300 were sufficient. In ex vivo vaginal tissue cultures, antigenic stimulation of T cells triggered antiviral responses in myeloid and CD4^+^ T cells, indicating that cellular immune responses may reduce the threshold of nAbs required for superior and durable protection. While these data were generated in macaques, they highlight the potential role of human CD4^+^ TRM in preventing or clearing infection and providing long-term protective immunity and suggest modalities for eliciting antigen-specific human TRM cells by vaccination. The rhesus CMV-SIV vaccine candidate, which is efficacious in macaque-SIV models acts through atypical MHC-I-E and MHC-II restricted CD8^+^ T cell activity and they can be shown at genital sites of challenge. An interaction with CD4^+^ TRMs is possible but not reported [263]. Accordingly, the induction of CD4^+^ and CD8^+^ TRM cells is a promising approach for designing effective vaccines against both HIV and HSV and may require specific adjuvants/chemokines to induce both CD4^+^ and CD8^+^ TRM cells.

## 8. Conclusions

Understanding the biology of CD8^+^ and CD4^+^ TRMs and their interactions in their various tissue niches will advance the development of vaccines and immunotherapy. For HIV and HSV-1/2, this is particularly important in the anogenital tract. For HSV-2, CD4^+^ and CD8^+^ TRMs are established after initial infection and then play a key role in controlling subsequent recurrences. However, CD4^+^ TRMs are also an inadvertent target for HIV infection. Detailed studies of HIV/HSV-2 interactions with CD4^+^ and CD8^+^ TRM may help devise strategies for counteracting the enhanced susceptibility to HIV acquisition that is mediated by HSV-2 infection. For both viruses, novel strategies to stimulate induction and maintenance of CD4^+^ and CD8^+^ TRM specific for HIV or HSV-2 might also lead to long-lasting local protection. Such approaches will require careful studies of CD8^+^ and CD4^+^ TRMs in human anogenital tissue explants as well as comparisons to non-human primates and possibly humanised mice as appropriate animal models for vaccine development.
